# Novel Blood Clot Retriever for Ischemic Stroke

**DOI:** 10.3390/mi12080928

**Published:** 2021-08-03

**Authors:** Ming-Ya Hung, Chun-Kai Yang, Jiong-Hong Chen, Li-Han Lin, Hao-Ming Hsiao

**Affiliations:** Department of Mechanical Engineering, National Taiwan University, Taipei 10617, Taiwan; d04522001@ntu.edu.tw (M.-Y.H.); r06522814@ntu.edu.tw (C.-K.Y.); r06522827@ntu.edu.tw (J.-H.C.); d09522010@g.ntu.edu.tw (L.-H.L.)

**Keywords:** ischemic stroke, blood clot retriever, medical device, Nitinol alloy, finite element analysis

## Abstract

Stroke is the second leading cause of death in the world. Ischemic stroke, caused by the blockage of intracranial arteries, accounts for approximately 80% of strokes. Among this proportion, acute ischemic stroke, usually caused by the sudden formation of blood clots, can cause fatal blockages in arteries. We proposed a unique blood clot retriever for the treatment of acute ischemic stroke, and conducted a series of tasks, including design, computer simulation, prototyping, and bench testing, for the proof of concept. Unlike most blood clot retrievers used today, our novel design deviates from traditional stent-like blood clot retrievers and uses large closed cells, irregular spikes, and strut protrusions to achieve maximum entanglement for better retrieval performance. Experimental results showed that the retrieval rate of our blood clot retriever was 79%, which demonstrated the feasibility of our new design concept.

## 1. Introduction

Stroke is the second leading cause of death worldwide. There are two types of stroke, namely ischemic and hemorrhagic stroke [1]. One cause of ischemic stroke is cerebrovascular occlusion, such as thrombosis, whereas hemorrhagic stroke is the rupture of cerebrovascular arteries [2]. Statistics show that up to 80% of strokes result from ischemic vascular occlusions. The numerous complications due to ischemic stroke can lead to long-term disability or death [3].

The current treatment for acute thrombosis, recombinant tissue plasminogen activator (rt-PA), utilizes drug injection into the human body to dissolve the occluding thrombus and recanalize the cerebrovascular arteries. However, some patients cannot be treated with rt-PA due to their specific medication conditions, so its practicality in clinical practice is limited. Therapy with rt-PA also increases the chance of bleeding complications; therefore, the clinical effect of intracranial artery occlusion requires further evaluation [4]. One study even shows that only 10% of ischemic stroke patients are eligible for thrombolysis treatments due to the hemorrhage risk and time constraints [5].

Another treatment option, mechanical thrombectomy, is achieved by interventional surgery involving percutaneous puncture, image guidance along the blood vessel to the lesion, and delivery of a retrieval device. Mechanical thrombectomy devices comprise a wide array of endovascular tools for removing blood clots in acute ischemic stroke patients. Each type of mechanical thrombectomy device achieves recanalization through somewhat different design mechanisms (e.g., coils, suction devices, stent retrievers). Among them, stent retrievers (also called blood clot retrievers) have gained increasing popularity in recent years. The combination of rt-PA drugs and stent retrievers could extend the prime time to six hours, leading to a lower bleeding rate and minimizing stroke complications [6]. Due to the unique properties of super-elastic nickel–titanium (NiTi) alloy, which is also known as Nitinol, the stent retriever can be crimped into a microcatheter for delivery and then spring back to its target diameter after release to engage with the blood clots. Nitinol is a commonly used material in the medical device industry and is approved by the FDA for many clinical applications. For example, peripheral stents are perhaps the most celebrated application of the Nitinol material. They are crush recoverable and more physiologically compatible than balloon-expandable stents in many indications such as carotid, superficial femoral, popliteal, and iliac arteries [7,8,9].

There are several commercial stent retriever products available on the market for clinical use today [7,8]. Solitaire (Medtronic, Minneapolis, MN, USA), with the stent-like look, is the most regularly used stent retriever in recent clinical trials. Trevo (Stryker, Kalamazoo, MI, USA), with a tapered distal end and closed-cell design, provides a smooth transition and better blood clot integration. ERIC (MicroVention, Tustin, CA, USA) adds spherical Nitinol wire cages in its design, improving access in challenging patient anatomy and reducing fragmentation of the blood clots. Several recent retriever studies from academia have proposed different concepts in designs and materials. Chon et al. built a retriever prototype using localized radio frequency (RF) to perform blood clot engagement with minimal stenting force [9]. Muschenborn et al. evaluated the feasibility of utilizing shape memory polymer (SMP) acrylates for a retriever device through FEA modeling [10].

Most existing blood clot retrievers maintain the traditional stent design or directly use currently available vascular stents [7,8]. The occluded blood vessel becomes recanalized after blood clots are extracted from the host’s body by the above-mentioned methods. Currently, two major retrievers commonly used in the medical field are coils and stent retrievers [11]. Coils are often formed of wrapped loops bound with a guidewire at one end to extract blood clots. Stent retrievers feature a mesh design and grids to engage with blood clots, and they are considered good candidates for blood clot retrievers [12].

This study aimed to design a blood clot retriever made especially for blood clot removal based on clinical needs. Due to its unique geometry, our designed stent retriever can engage with blood clots to achieve the highest blood clot removal rate. In this study, the development of the novel blood clot retriever was evaluated by finite element analysis [13,14], laser manufacturing, and in vitro clot retrieval tests [9]. The finite element models were developed to analyze the mechanical behavior and manufacturing feasibility of the devices. Prototypes of blood clot retrievers were then produced by laser cutting. The results of in vitro clot retrieval tests provide evidence for the proof of concept. We expect this novel design to bring a great leap forward in blood clot removal and provide stroke patients a better and newer choice.

## 2. Materials and Methods

### 2.1. Blood Clot Retriever Design

**Figure 1:**
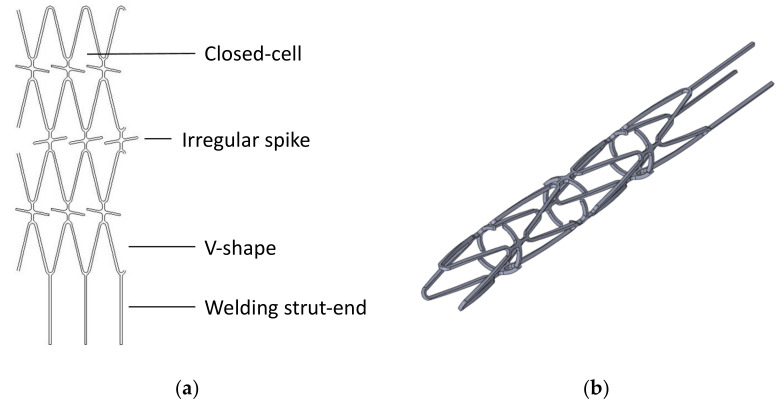
A design concept of a novel blood clot retriever: (**a**) 2D sketch of the blood clot retriever with irregular spikes and V-shaped hollow closed cells; (**b**) 3D view of the blood clot retriever.

In the design of a blood clot retriever, the most important clinical need is the ability of the retriever to remove blood clots. The blood clot retriever and the blood clots need to be tightly engaged to achieve an ideal blood clot removal rate. Our design concept is based on a large, hollow closed cell with a V-shaped design, which traps the blood clot deeply in the closed cell such that it cannot easily become dislodged; the irregular protrusions, which are similar to spikes, can engage a blood clot and tightly fix it in the blood clot retriever (Figure 1a). Figure 1b is a 3D view of the novel blood clot retriever showing that it has a unique geometry with a concave–convex surface. Compared with the simple, smooth surface of the traditional vascular stent, the novel blood clot retriever can increase the removal rate with these unique irregular spikes and large closed cells.

In this study, Solidworks software (Dassault Systems Solidworks Corp., Waltham, MA, USA) was used to design the blood clot retriever. The mathematical relationship between geometric parameters was established in a previous study [15], which allowed quick modification of the geometry of the blood clot retriever and efficient optimization of the design. After the drawn sketch was imported into the finite element solver, the iterative design process was continued until the simulation performance met the mechanical requirements.

### 2.2. Finite Element Analysis

In this study, the ABAQUS/Standard finite element solver (Dassault Systèmes Simulia Corp., Providence, RI, USA) was used to create various finite element models to simulate the steps of the blood clot retriever being manufactured and crimped into the catheter [16,17].

#### 2.2.1. Material Properties

**Figure 2:**
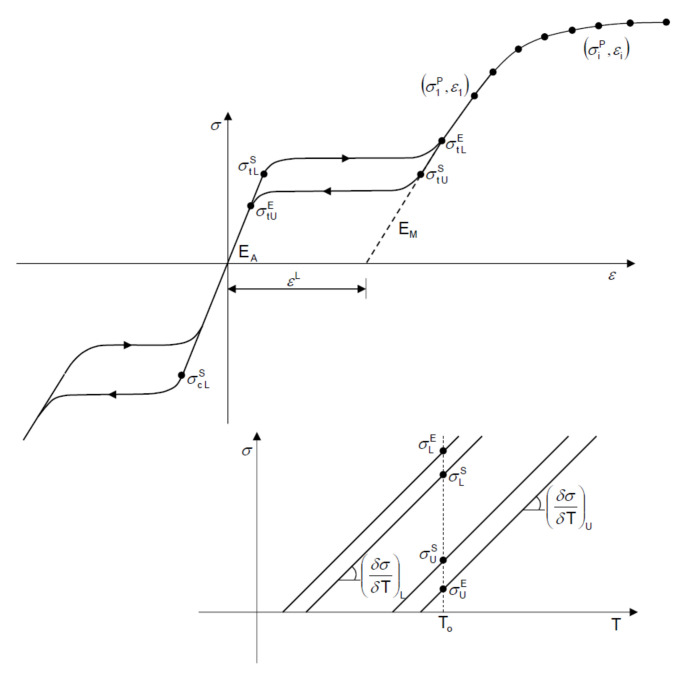
The stress–strain curve of the Nitinol used in the ABAQUS UMAT (user-defined material) subroutine.

The properties of Nitinol were chosen based on the literature [18]. Figure 2 shows the super-elastic stress–strain curve of this material. The material parameters in the FEA models were set using the ABAQUS UMAT (user-defined material) subroutine. The different state points of the Nitinol stress–strain curve were applied to the finite element model to simulate the material behavior of Nitinol.

#### 2.2.2. Manufacturing Simulation

Finite element simulation was used to evaluate whether the designed blood clot retriever met clinical needs. The computer simulation was performed with the following steps:

Step 1: Expand the 2.0 mm diameter blood clot retriever to 4.5 mm in diameter.
Step 2: Anneal the blood clot retriever to eliminate residual stress.
Step 3: Weld the strut end.
Step 4: Crimp the blood clot retriever into a 1.4 mm diameter catheter.

The simulated model of the blood clot retriever was placed in a cylindrical coordinate system (r, θ, z). There were three parts: the blood clot retriever itself and two cylindrical sleeves placed inside and outside the stent. The inner and the outer cylindrical sleeves were applied to expand and crimp the blood clot retriever, respectively. For the selection of an element, the blood clot retriever was assigned the cubic element C3D8I, while the surface element SFM3D4R was used for the cylindrical sleeves.

### 2.3. Materials and Prototype Manufacturing

The blood clot retriever was manufactured in four steps: laser cutting, annealing, sandblasting, and electrolytic polishing.

#### 2.3.1. Laser Cutting

**Figure 3:**
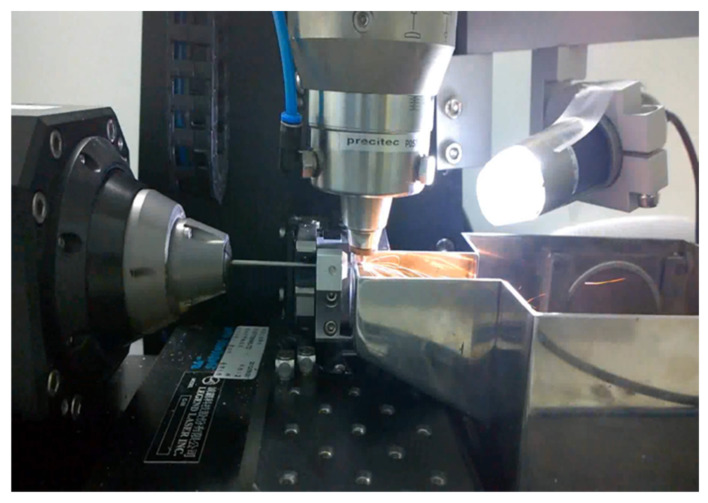
A 100 W fiber laser module comprising an Aerotech linear X–Y motor stage and a Z-direction servo motor.

A laser module consisting of a Rofin 100 W pulsed fiber laser, an Aerotech linear X–Y motor stage, and a Z-direction servo motor were assembled and integrated (Figure 3). In the laser cutting module, the 2D sketch was imported and then converted into a 3D laser cutting coded path. The coded path was cut onto a seamless Nitinol hypotube with a diameter of 2.0 mm (Minitubes, Grenoble, France).

#### 2.3.2. Annealing and Shaping

Due to the super-elastic properties of Nitinol, a stent can automatically expand to a large target size when released from a small catheter. To create such super-elastic effects, annealing treatments were applied after the laser cutting. The annealing process assisted with the shaping and also eliminated the residual stress resulting from expansion. The expansion was completed by the insertion of a cone steel rod with the desired diameter. After expansion, a blood clot retriever with steel rods was placed in a salt bath furnace at 500 °C for 200 s. The high temperatures associated with laser cutting and annealing might create spatter, oxide layers, and other debris that would be removed by further processing.

#### 2.3.3. Sandblasting and Electrolytic Polishing

The surface finishing was divided into two major steps: sandblasting and electropolishing. Sandblasting was first applied with alumina particles (28–32 μm) sprayed at a pressure of 2 kg/cm^2^ for one minute to remove large debris. Electropolishing was then conducted to deburr the surface and remove small defects. The electropolishing solution was mixed with 21% (volume) perchloric acid and 79% (volume) acetic acid. The anode and the cathode were made of stainless steel flakes and titanium wire, respectively. With a voltage of 5.2 volts, a mirror-like surface could be achieved at room temperature in 90 s.

### 2.4. In Vitro Bloot Clot Removal Test

This test was performed to visually evaluate the ability of the blood clot retriever to maintain the engagement of the blood clots within the struts during the retrieval [19]. The blood vessel part was a clear acrylic tube with an inner diameter of 4.0 mm. Jelly (gel network material formed by pectin, citric acids, and sugar) was injected into the acrylic tube to simulate a blood clot inside a blood vessel. The blood clot retriever was crimped by a microcatheter (steel pipe with an outer diameter of 2.0 mm), delivered across the blood clot (jelly) of the vascular model, and deployed against the blood clot following the current clinical practice. The Nitinol blood clot retriever expanded automatically once the steel pipe was retracted. The blood clot retriever with the jelly was then gently pulled back. The measured weights of the acrylic tube before and after the experiment were calculated with Equation (1):

Blood clot removal rate % = ((Weight before removal − Weight after removal)/Weight before removal) × 100%(1)

where the weight before removal is the blocked blood vessel (injected tube with jelly), while the weight after removal is the empty acrylic tube. The blood clot removal rate, determined by comparing the weight percentage of the removed jelly, indicated the efficiency of the blood clot retriever.

## 3. Results

### 3.1. Novel Blood Clot Retriever Design

**Figure 4:**
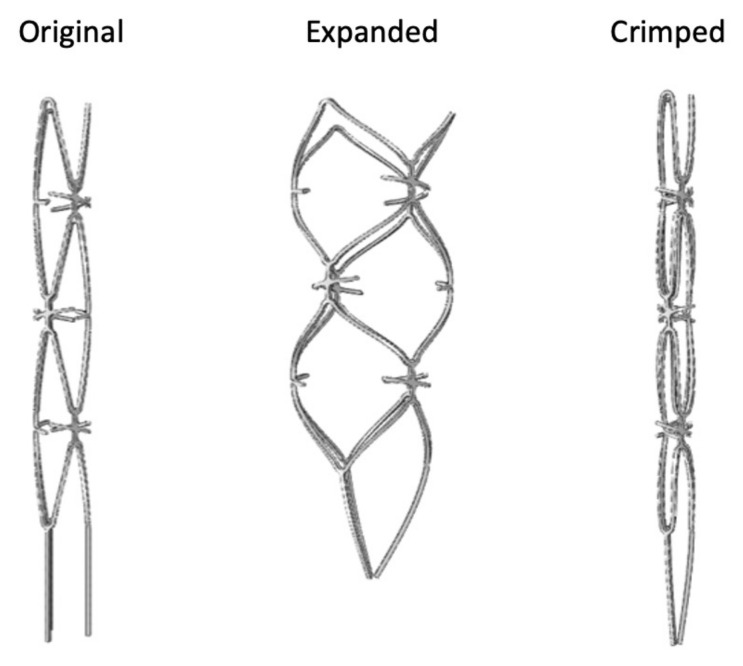
Geometric profiles of the novel blood clot retriever in the original (**left**), expanded (**middle**), and crimped (**right**) states.

Figure 4 shows the geometric changes in the blood clot retriever during expansion and crimping. During the expansion, the large closed-cell design produced unique geometry with a concave–convex surface, as expected, which could be seen from the side view. This feature also promoted tighter engagement of the blood clot retriever with the blood clots. Moreover, the irregular protrusions (little spikes) acted as complicated traps in each cell that could help tightly fix the blood clots. With this design, once the blood clot is trapped deep in the closed cell, it cannot easily become dislodged and escape. Compared with the traditional vascular stent, the novel blood clot retriever with these unique irregular spikes and large closed cells could increase the removal rate.

### 3.2. Finite Element Analysis

**Figure 5:**
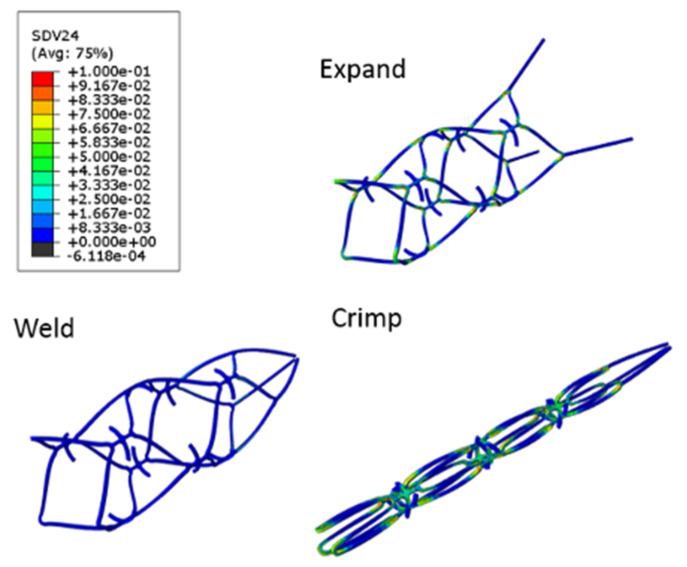
Contour plot comparison of plastic strain in the novel blood retriever in the expanded (**top**), welded (**bottom left**), and crimped (**bottom right**) states.

Nitinol has a special property, namely, a 12% maximum tensile strain threshold for the shape recovery mechanism. Under the upper bound limit, the stent design is forced to cooperate with this strain limit during manufacturing. The results shown in Figure 5 indicated that the maximum strain distribution of the blood clot retriever during the entire manufacturing process was below the 12% strain limit. The computer simulation also showed the geometric changes of the blood clot retriever during expansion and crimping. The contours of the large closed cells produced an obvious concave–convex surface as well. We expected this geometric change to help the blood clot retriever engage with the blood clots more tightly during further deployment and self-expansion.

### 3.3. Laser Cutting

**Figure 6:**
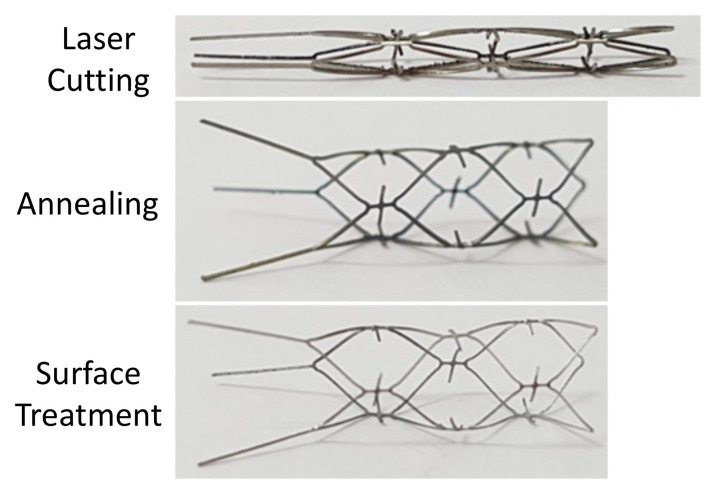
The prototype of the novel blood clot retriever after laser cutting (**top**), annealing (**middle**), and surface treatment (**bottom**).

Figure 6 shows the prototype product after laser cutting, annealing, and surface treatments (sandblasting and polishing). Laser cutting was used to engrave the original design pattern onto a Nitinol hypotube with a diameter of 2.0 mm. The blood clot retriever was successfully expanded from a diameter of 2.0 mm to the target size of 4.5 mm by annealing and shaping. Finally, the debris and burrs were removed by surface treatments, including sandblasting and polishing, to achieve a mirror-like surface. The prototype of the blood clot retriever is presented in Figure 5 as our design concept.

### 3.4. In Vitro Retrieval Test

**Figure 7:**
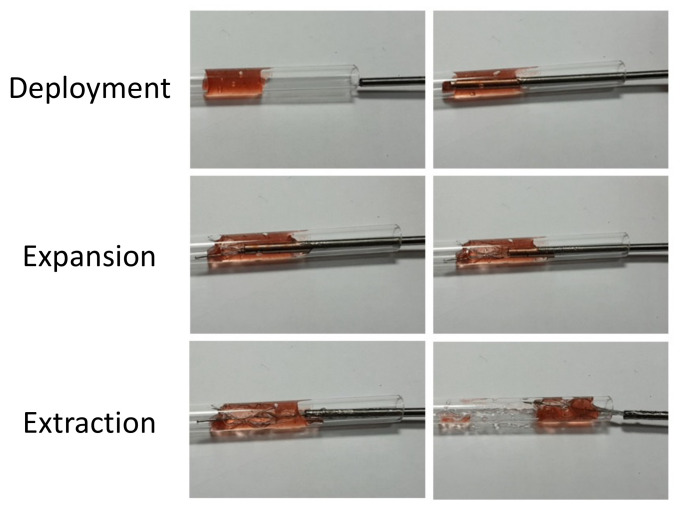
In vitro test of the novel blood clot retriever during deployment, expansion, and extraction.

This test visually evaluated the retrieval ability during the recanalization process, including deployment, expansion, and blood clot extraction. After jelly was injected into an empty acrylic tube to create a blockage of 1.5 cm in length, the welded blood clot retriever was crimped into a steel pipe and transported into the injected acrylic tube by a guidewire. This step also ensured the ability of the retriever to be crimped into a 2.0 mm catheter. The complete retrieval process is shown in Figure 7. First, the blood clot retriever was pushed within a catheter (steel pipe) through the blood clot (jelly), and then the catheter was retracted to allow the blood clot retriever to self-expand. The blood clot (jelly) could be firmly trapped in the large closed cell of the blood clot retriever. Finally, the guidewire welded to the strut end was used to retract the retriever from the acrylic tube.

**Figure 8:**
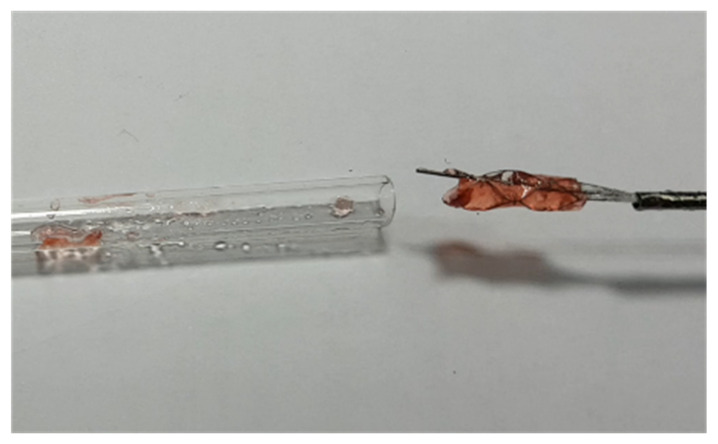
In vitro test of the novel blood clot retriever after retraction.

The measured weights of the empty acrylic tube before and after the experiment were calculated. The results shown in Figure 8 revealed that most of the jelly was attached to the blood clot retriever and retracted from the acrylic tube successfully, while only a small amount of the jelly remained attached to the wall of the tube. The retrieval test was repeated seven times. The results and calculated retrieval rate, shown in Table 1, indicated that the average retrieval rate was 79%. The test sufficiently proved that our novel blood clot retriever with a unique geometric design had a good retrieval rate.

## 4. Discussion

Although this novel blood clot retriever proved its good ability with a 79% retrieval rate, there are two limitations for discussion. Firstly, the in vitro tests conducted in this study used jelly and acrylic tubes to simulate blood clots and blood vessels. This was insufficiently ideal, as both the jelly and acrylic tubes had simple homogeneous properties, as opposed to the more complicated blood clots and blood vessels, which are heterogeneous in nature. If real blood was used, blood clot coagulation would occur after a long period outside the host body, so appropriate amounts of anticoagulants are required to control the coagulation rate. However, this aspect was beyond the scope of our study. In this study, we aimed to prove the feasibility of our retriever design, so a more systematic comparison under a controlled and observable environment was preferred. The jelly and acrylic tubes offered less complicated conditions, which helped us to reduce the testing variables associated with blood clots and blood vessels. Liebig et al. presented a pulsatile flow model to simulate the blood clot retrieval process. A transparent acrylic tube was used as the blood vessel model for observation [19]. Wenger et al. proposed a vascular glass model to evaluate the efficacy of the stent retriever design [20]. Chon et al. used a transparent Tygon tube to simulate a blood vessel in their in vitro testing [9]. In our study, a transparent acrylic tube, similar to Liebig’s model, was used, and the retrieval process could be observed and recorded easily. In the future, we plan to use actual porcine blood with anticoagulants to mimic a more realistic in vitro environment.

Second, due to the limitations of our lab environment, the blood clot retrieval rate measured in this study was different from the recanalization rate used in clinical trials. The recanalization rate is defined as achieving Thrombolysis in Myocardial Infarction (TIMI) flow in all treatable vessels, which is beyond the scope of our study [21]. The TIMI score defines the vessel patency (residual clot burden) by angiography and flow grade (grade 1–2 flow, occluded; grade 3–5 flow, successful recanalization, determined by the rate of blood flow) [22]. One of the mechanical thrombectomy clinical trials showed that the first-generation MERCI device achieved a recanalization rate of only 48%, and, when coupled with intraarterial thrombolytic drugs, the recanalization rate improved to 60%. Our scoring system, based on weight measurements, is considered more stringent than the recanalization rate, in which partial blood flow with residual clots can be scored. Test results showed that the acrylic tube after retrieval was almost clear and empty. Our blood clot retrieval rate of 79% is higher than the 60% of MERCI with thrombolytic drugs, which proves our concept and demonstrates the feasibility of our retriever design. Another two commercial blood clot retrievers with traditional stent appearances, Solitaire and Trevo, often produce micro- and macro-fragments of blood clots during penetration and retrieval. Although these stent retrievers have acceptable retrieval rates, their dislodgement of blood clots is higher than desired during procedures. Our design showed little dislodgement of blood clots in the in vitro test due to its unique design of irregular spikes and strut protrusions.

**Figure 9:**
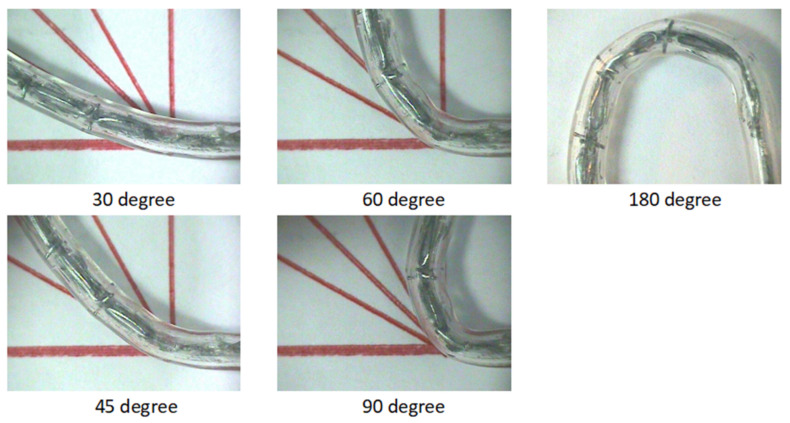
Bending test of the novel blood clot retriever.

Retriever deliverability is an important clinical attribute, as delivery failure occurs sometimes in percutaneous coronary interventions (PCI) and most of these failures are due to vessel tortuosity. Deliverability is described as the flexibility of a retriever in its crimped state with adaptation to the natural shape of the vessel. A retriever design with excellent flexibility could potentially shorten PCI procedure times in the technically challenging subgroup of patients. A series of bending tests were performed to demonstrate the flexibility and thus the deliverability of our retriever design. The retriever was inserted inside a tube and bent at 30°, 45°, 60°, 90°, and 180° angles. Test results showed that our retriever design was able to comply with the tubing geometry while bending (Figure 9).

In future work, our research will focus on more complicated vasculature systems, such as bifurcations or curved blood vessels, to further validate the feasibility of our retriever design under various conditions. We also plan to use porcine blood with anticoagulants to mimic a more realistic in vitro environment.

## 5. Conclusions

This research realized the design concept of a novel blood clot retriever for ischemic stroke and completed a series of tasks, namely design, computer simulation, prototyping, and experimental verification. Having a large closed-cell design and irregular protrusions similar to spikes, this novel blood clot retriever could deeply engage with blood clots and thus achieve excellent blood clot removal rates. We employed laser cutting, annealing, sandblasting, and electrolytic polishing to manufacture the prototype. A complete proof of concept was therefore demonstrated and in vitro bench tests were then evaluated. Experimental results showed that the average retrieval rate of this novel blood clot retriever was 79%, which proved our concept and demonstrated the feasibility of our retriever design. It is a great leap forward for blood clot retrieval and could provide stroke patients with another choice in the types of surgery available.

## Figures and Tables

**Table 1:** In vitro retrieval test. The weight of the empty acrylic tube was 1.36 g.

Weight before Removal (g)	Weight after Removal (g)	Retrieval Rate %
1.538	1.378	89
1.546	1.419	68
1.566	1.402	80
1.557	1.385	87
1.568	1.409	76
1.567	1.394	84
1.552	1.423	67

## Data Availability

The data used to support the findings of this study are included within the article.
